# Conservation units alone are insufficient to protect Brazilian Amazonian chelonians

**DOI:** 10.1038/s41598-024-61722-y

**Published:** 2024-05-11

**Authors:** Loyriane Moura Sousa, Letícia Lima Correia, Rafaela Jemely Rodrigues Alexandre, Simone Almeida Pena, Thiago Bernardi Vieira

**Affiliations:** 1 Programa de Pós-Graduação em Zoologia, Universidade Federal do Pará, Campus de Belém, Rua Augusto Corrêa, No 01, Guamá, 66075-110 Brazil; 2 Programa de Pós-Graduação em Ecologia, Universidade Federal do Pará, Campus de Belém, Rua Augusto Corrêa, No 01, Guamá, 66075-110 Brazil; 3 Laboratório de Ecologia, Faculdade Federal do Pará, Campus de Altamira, Rua Coronel José Porfirio, No 030, Altamira, PA Brazil

**Keywords:** Conservation unit, Indigenous lands, Biodiversity, Bioclimatic variables, Turtle, Tortoise, Biodiversity, Ecological modelling, Conservation biology

## Abstract

The creation of protected areas (PAs) is not always based on science; consequently, some aquatic species may not receive the same level of protection as terrestrial ones. The objective of this study was to identify priority areas for the conservation of chelonians in the Brazilian Amazon basin and assess the contribution of PAs, distinguishing between Full Protection Areas, Sustainable Use Areas, and Indigenous Lands for group protection. The entire species modeling procedure was carried out using Species Distribution Models. Location records were obtained from platforms such as SpeciesLink, GBIF, the Hydroatlas database, and WorldClim for bioclimatic variables adjusted with algorithms like Maximum Entropy, Random Forest, Support Vector Machine, and Gaussian-Bayesian. Indigenous lands cover more than 50% of the distribution areas of chelonian species in the Brazilian Amazon. Protected areas with higher conservation importance (Full Protection Areas and Sustainable Use Areas) hold less than 15% of the combined species distribution. Researchers face significant challenges when making decisions with models, especially in conservation efforts involving diverse taxa that differ significantly from one another within a group of individuals.

## Introduction

Global biodiversity is under anthropogenic pressure, and in many cases, it is being lost even before being fully understood; many animals are at risk of extinction without in-depth knowledge of their species^1,2^. This phenomenon is occurring due to significant technological advancements and the construction of major developments that impact river courses; as a result, aquatic species have experienced a heightened decline^3,4^. All of these factors accelerate the loss of diversity through changes in land use and cover, and this is observed in the Brazilian Amazon for different taxonomic groups, such as amphibians^5^, birds^6^, bats^7^ and fish^8^. The main causes of changes in land use and coverage in the Amazon are the expansion of agricultural frontiers, mineral extraction, logging and, in the main rivers, the construction of hydroelectric plants^9,10^. Considering the deforestation of riparian areas and the construction of hydroelectric plants as the main causes of biodiversity loss in Amazon rivers^11^.

Turtles play a very important socio-economic role in the Amazon region. Indigenous people and riverine communities have been fishing and trading these animals for generations^12^. Among the consumed species, the Amazonian turtles most traded and used for consumption belong to the genus *Podocnemis*^13^. In addition to being considered important for protein consumption, their eggs were historically extracted for oil, used as fuel for public lighting during the colonial period^14^. These animals play a crucial role in the functioning of various ecosystem services, especially in the food web, seed dispersal, and organic matter cycling^15^. Amazonian turtles have experienced a significant population decline due to factors such as large hydroelectric dam constructions in watercourses, resulting in increased water pollution, agricultural activities, deforestation of riparian and floodplain areas, river damming, bodies of water, and illegal consumption and trade of turtles^16,17^.

In addition to the lack of knowledge about taxonomic diversity (Linnean deficit), especially in more remote areas like the Amazon, there are uncertainties or even total ignorance about the geographical distribution of species (Wallacean deficit)^18–20^. These two gaps pose significant challenges for biodiversity conservation, especially when areas of economic interest overlap with those of high biological conservation value^21^. Therefore, the lack of knowledge about the geographical distribution of species, combined with the unavailability of financial resources and the overlap with areas of economic interest, represents the main challenges for conservation^22,23^.

One way to mitigate biodiversity loss is through research on the species found in specific areas, determining their significance for these animals, and subsequently implementing conservation areas^22^, known as Protected Areas (PAs) in Brazil^24^. The creation of a PAs normally occurs when there is a social demand to protect areas of biological or cultural importance, or even to ensure the sustainable use of natural resources by traditional populations^24^. There are 12 recognized types of PAs distributed in two sets: 1. Full Protection Units (SPA), with the primary goal of maintaining ecosystems, allowing only the indirect use of their natural resources; and 2. Sustainable Use Units (SUA), aiming to reconcile nature conservation with the sustainable use of a portion of their natural resources^24^. In addition to these PAs categories in Brazil, there are Indigenous Lands (TIs), defined by the state as territories where native peoples can live in their natural environment^25^. According to Article 231 of the 1988 Constitution, these lands are permanently inhabited by indigenous peoples and can be used for housing and subsistence. The residents have permanent possession and exclusive use of their resources^26^. Although these lands are not considered PAs according to the National System of Conservation Units (SNUC), they are recognized as protected lands by the Brazilian government.

However, the effectiveness of PAs and ITs for biodiversity conservation has been questioned and investigated^27,28^. Mainly through processes called Downgrading, Downsizing and Degazettement, or PADDD, these changes can put ecosystems, species and people who depend on them at risk^29^. The location of these areas is arbitrarily defined based on economic and/or political interests, often in areas with low economic interest or based on researchers' empirical knowledge, mainly focusing on specific taxonomic groups. As a result, these PAs were not designed for aquatic organisms^30^. One way to address this issue would be to define the location of PAs through Systematic Conservation Planning^31^, whose goal is to identify areas with high importance for biodiversity conservation, considering the principles of complementarity and irreplaceability of these areas^32^.

Turtles, tortoises, and terrapins, belonging to the suborders Cryptorida and Pleurodira^33,34^ inhabit semi-aquatic, and terrestrial environments^35,36^. They have various feeding habits, including omnivorous, carnivorous, and herbivorous diets. Turtles consume a variety of foods such as fish, dead matter, plants, fruits, seeds, and insects, playing crucial roles in food webs^37^. In addition to their ecological roles, they contribute to important functions such as seed dispersal^36,38^. Commonly known as “bichos de cascos” in the Brazilian Amazon, turtles are hunted, fished, and consumed by numerous residents of the Brazilian Amazon^37,39^. Considered one of the most endangered vertebrates^40^, over 50% of the 356 known turtle species^41^ are listed to some degree of threat by the IUCN^40,42^.

Species distribution models, known as SDMs, are important for species conservation and management^28,43^. They produce environmental suitability maps of species in areas that have not been previously sampled^23,44^, helping, for example, to update the distribution of current species, identify potential new areas for the occurrence of species, contribute to the IUCN red list and predict the potential impact of anthropogenic effects^45^. Therefore, these models are crucial for evaluating protected areas^46,47^. Gap analyzes show that protected areas (PAs) in the Americas are not suitable for biodiversity conservation^48,49^.

Therefore, our objective was to identify the contribution of PAs, differentiating between SPA and SUA, and TIs to the protection of Amazonian turtles. Even though TIs are not designated for biodiversity conservation, they significantly influence this process, as indigenous peoples require greater environmental integrity in their areas^30^. Additionally, we will identify priority areas for the conservation of turtles in the Brazilian Amazon basin and assess if PAs and TIs are in these high-importance areas. These procedures will be conducted considering all turtle species occurring in the Brazilian Amazon basin and subsequently for different IUCN conservation categories, as well as for habitats (terrestrial and semi-aquatic) and environments (generalists, lentic, lotic, and terrestrial).

## Results

**Figure 1:**
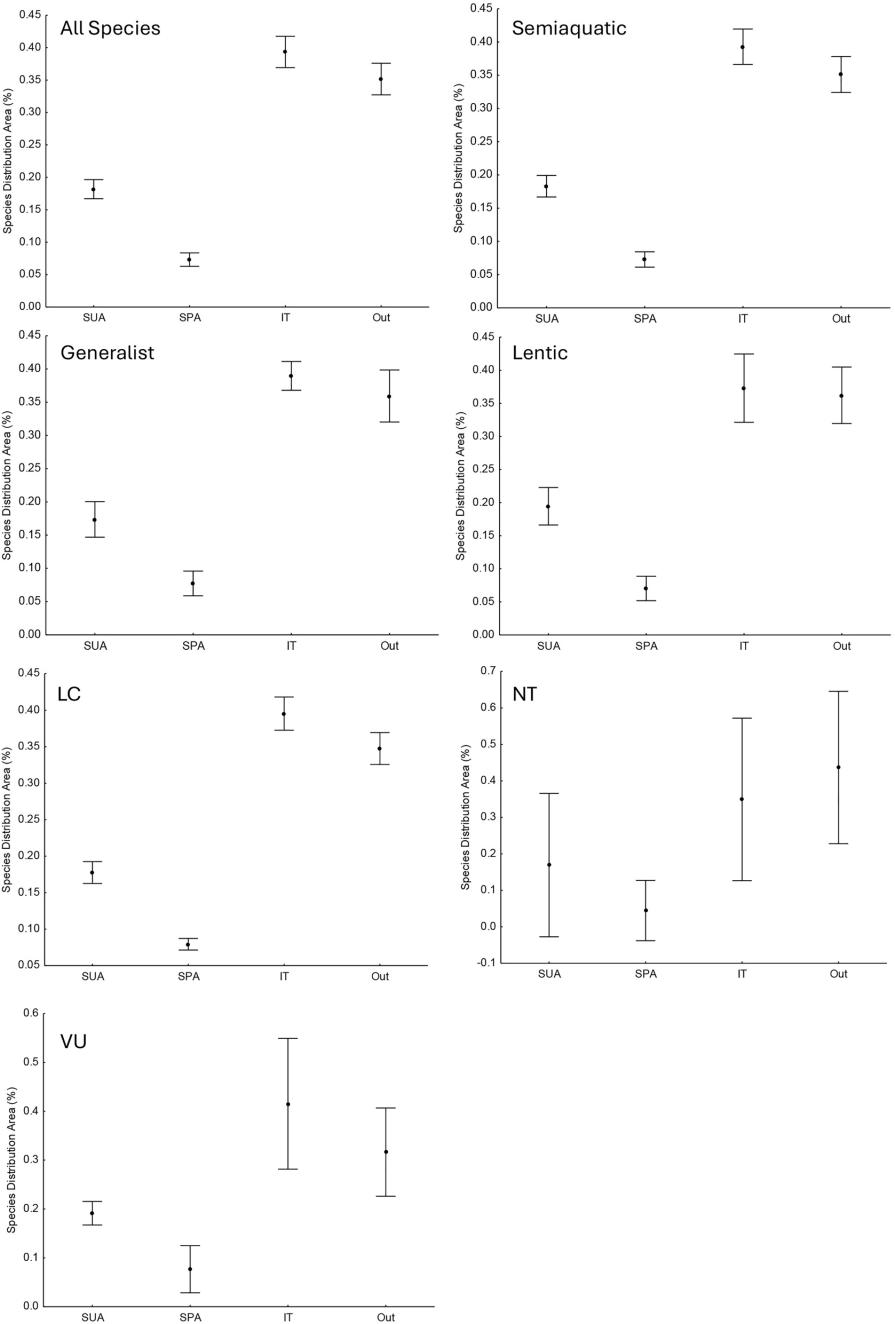
Analysis of Variance (ANOVA) for Percentage (Perc) of the distribution area of chelonian species with occurrences in the Brazilian Amazon basin concerning the type of conservation unit. The analyses were performed for the total distribution area by habitat environment and conservation status according to IUCN and their occurrences in PAs, ITs and outside these units. Outside—Outside of PAs or IT; IT—Indigenous Land; SPA—Full Protection Unit; SUA—Sustainable Use Unit; LC—Least Concern; VU—Vulnerable; NT—Near Threatened; IUCN—International Union for Conservation of Nature.

**Figure 2:**
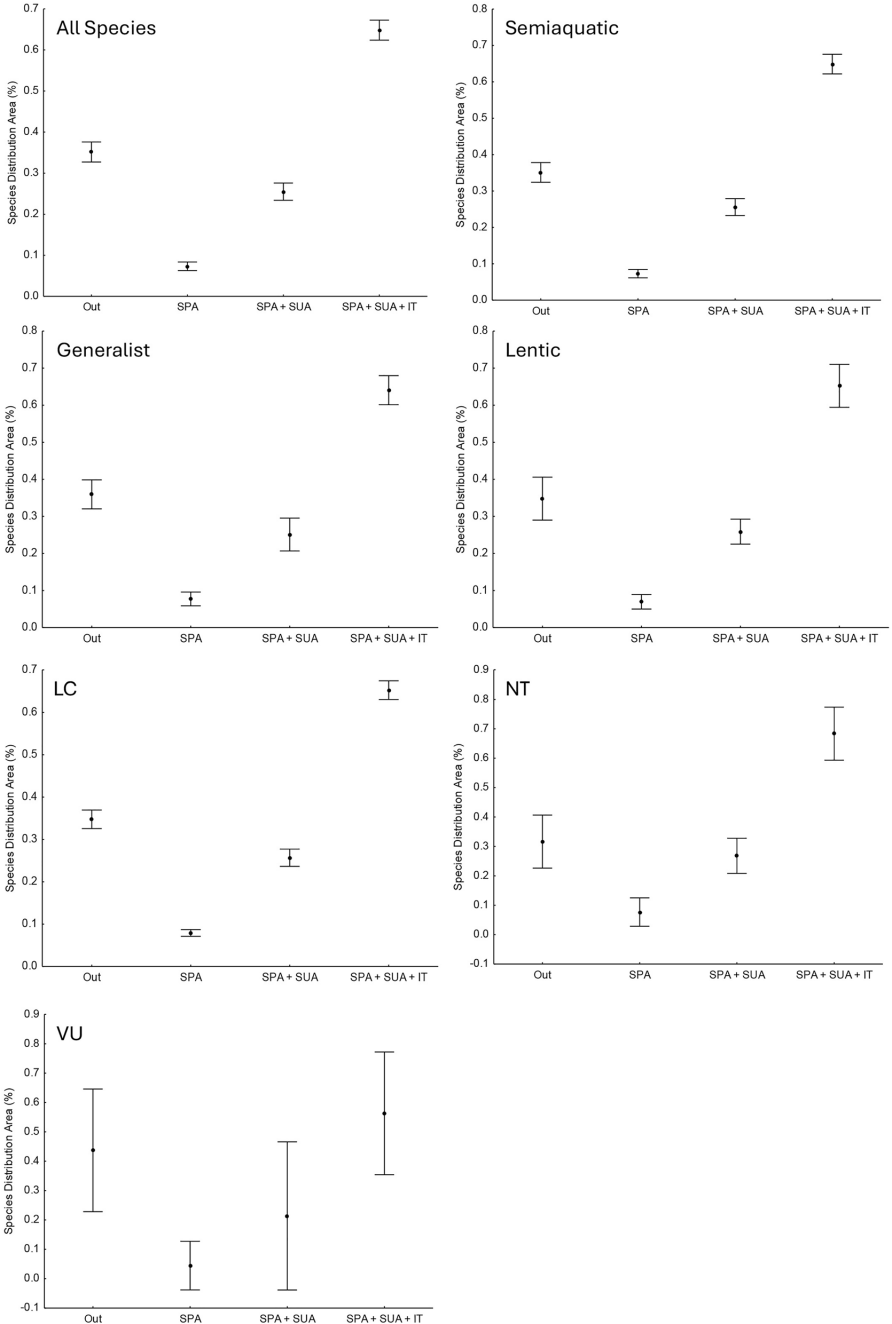
Analysis of Variance (ANOVA) for Cumulative Percentage (Perc Acc) of the distribution area of chelonian species with occurrences in the Brazilian Amazon basin concerning the type of conservation unit. The analyses were performed for the total distribution area by habitat environment and conservation status according to IUCN and their occurrences in PAs, ITs and outside these units. Outside—Outside of PAs or IT; IT—Indigenous Land; SPA—Full Protection Unit; SUA—Sustainable Use Unit; F—Fisher's F; df—Degrees of Freedom; p—Probability of Type I Error; LC—Least Concern; VU—Vulnerable; NT—Near Threatened; IUCN—International Union for Conservation of Nature.

We observed that approximately 35% of the entire geographical distribution of turtle species in the Brazilian Amazon is outside Protected Areas (PAs) and Indigenous Lands (ITs) Fig. 1). Within the conservation units (Full Protection Units—SPA and Sustainable Use Units—SUA), we found that SUA has the highest percentages of species occurrence areas (Fig. 2). However, ITs are the areas with the highest average percentages of distribution areas, with values exceeding 39% (Fig. 1). SPAs showed the lowest percentage values of distribution area, with values below 7% (Fig. 1). This pattern, with higher percentages in ITs and lower in PAs (SPA and SUA), becomes more evident in the analysis of cumulative percentage of distribution area. Only with the addition of ITs, the species reach values above 39% protected, with an average close to 64% (Fig. 2).

**Figure 3:**
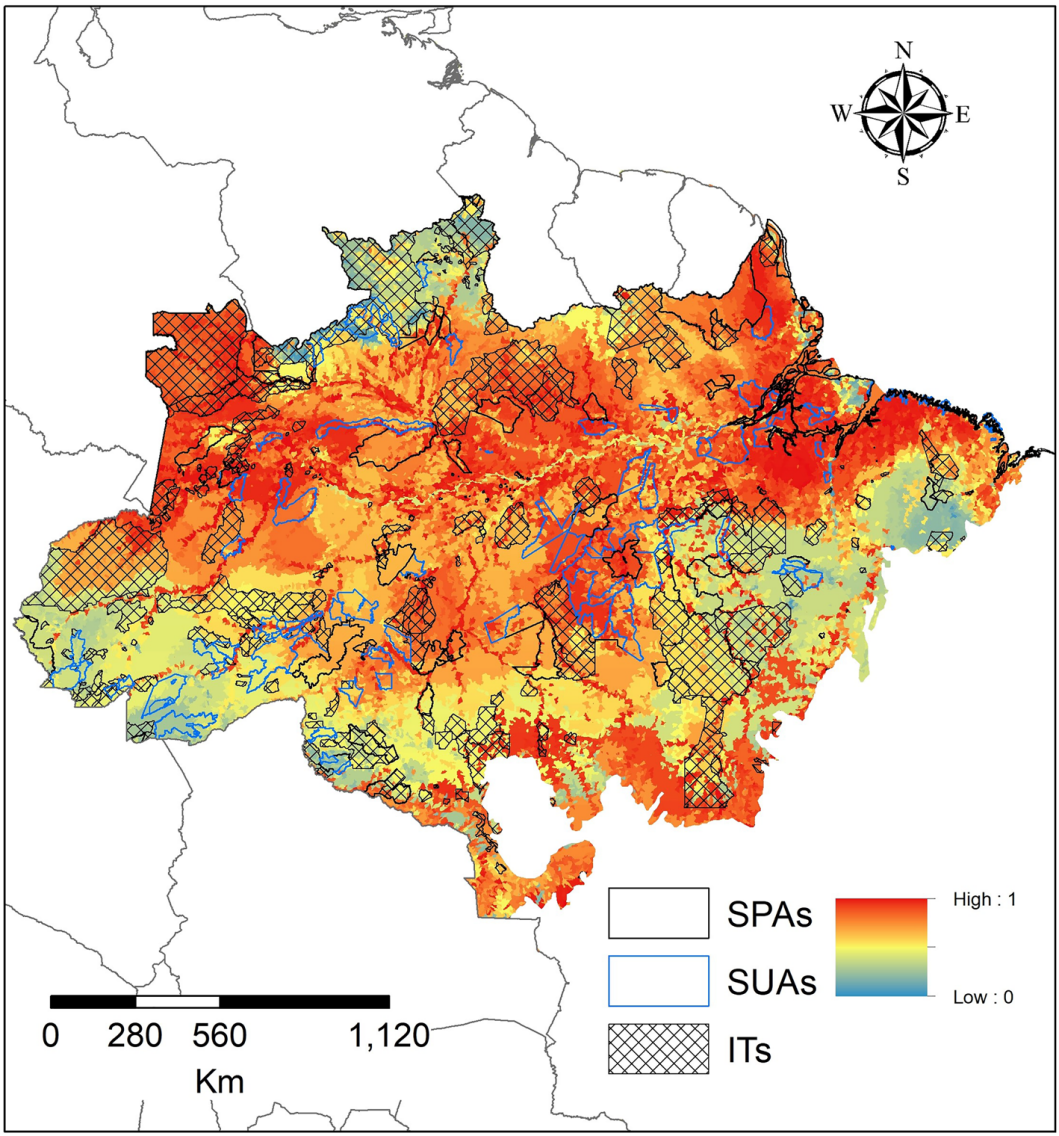
Priority areas for the conservation of chelonians with distribution in the Brazilian Amazon. To create the figures, the Qgis software version 3.34.3 (https://qgis.org/pt_BR/site/) was used.

The areas with greater importance for the conservation of turtle species in the Brazilian Amazon were concentrated in the northwest and northeast portions of the Brazilian Amazon basin (Fig. 3). This same pattern is observed for almost all groupings made, both for habitat and environment, and conservation status (Fig. 3), except for terrestrial environment (Fig. S01B), which showed more important areas in the southeast of the basin. The type of environment and IUCN classification can be seen in Figs. S02 and S03.

Regarding the importance of protected areas (PAs + ITs), we observed that the areas with higher importance for conservation are inside the SPA (Table S01). However, the ITs and SUAs show high importance for turtle conservation in the Amazon (Table S01). This same pattern, SPAs in areas with high importance values and ITs by chance, repeats for some groupings, such as terrestrial and semi-aquatic habitat turtles, generalist, classified as LC by and NT the IUCN (Table S01). For terrestrial species it was only significant with the addition of PAs + Its (Table S01).

## Discussion

These findings suggest that Indigenous Lands (ITs) play a crucial role in the conservation of turtle species in the Brazilian Amazon, contributing more than the conservation units (39%) of the distribution areas, but this contribution may be threatened by Marco Temporal—Law no 14,701/2023, which may affect the demarcation of indigenous lands.

However, when mapping priority areas for conservation, it is evident that ITs are over random areas, while protected areas, especially Full Protection Units (SPA) and Sustainable Use Units (SUA), have greater importance. This highlights a potential deficit in protection for the group, as designated protection areas tend to cover smaller portions of species distribution, totaling less than 15% combined, this number is below that recommended to guarantee the conservation of the group. Even with participatory management to preserve chelonians, one of the reasons for the decline of terrestrial and aquatic species is exploitation for food (traditional people or not), hunting and illegal trade^28,50,51^.

And one of the forms of conservation is protecting nesting beaches and establishing reserve areas where adult animals cannot be captured^52–54^. Which leads us to think that even with conservation units, it is important to carry out environmental education^55^, especially with people who are not residents of conservation units or indigenous land.

However, these areas have been experiencing events of loss of protected areas, a phenomenon referred to by researchers with the acronym PADDD (Protected Areas downgrading, downsizing, and degazettement). This phenomenon involves three different processes: reducing boundaries, complete elimination of protected areas, which relate to the pressures faced by these areas^29^.

In Brazil, the factors motivating this occurrence are anthropogenic activities as: agricultural constructions and large hydroelectric projects^56^. These events have been occurring more frequently in the Amazon biome since 2008. Of the 156 registered Protected Areas (PAs), 46 of them experienced the PADDD event from 1988 to 2018^56^, affecting 7.3 million hectares^57^. Fagundes et al. 2021, shows that only 11% of sandbanks have nesting sites confirmed by experts^53^. These factors will influence the reproduction and survival of chelonians, considering that these sandbanks are not within indigenous lands along with conservation units, as we saw in this work that the two together have a high influence of 64% for all species.

Tropical regions are highly diverse, and the taxonomic knowledge gap (Linnean Shortfall) is closely associated with the lack of understanding of factors influencing the geographical distribution of fauna across this heterogeneous domain (Wallacean Shortfall)^58^. Consequently, it becomes impossible to measure or represent all species, both terrestrial and aquatic. Thus, choices in modeling can play a crucial role in determining priority areas or regions. Systematic Conservation Planning (SCP) aims to assist stakeholders in decision-making by providing conservation protocols^31,59^. One of the significant challenges in conservation planning is the gaps in the distributions of numerous species and their geographical ranges, which are poorly known^58^.

We observed that the largest distribution areas of chelonians were within Indigenous Lands (more than 40% of occurrence points). Indigenous peoples rely on chelonians as a significant food source, consuming adults, juveniles, and their eggs, with the latter being the most consumed item^12^. Aquatic species such as *Podocnemis expansa* (Tartaruga-da-Amazônia) and *Podocnemis unifilis* (Tracajá) are the most consumed due to their higher abundance, broader distribution, and larger body size. However, these communities tend to have a more established relationship with these animals, respecting their reproductive cycle and limits^60^. Terrestrial chelonians species are also widely consumed by these populations, such as *Chelonoidis carbonarius* and *Chelonoidis denticulatus*^61^. However, inadequate supervision, illegal consumption, and trade of these chelonians, both within Indigenous Lands and Protected Areas, pose significant threats, particularly during the nesting phase when these animals are vulnerable on plateaus, beaches, riverbanks, and clustered near nesting sites throughout the Brazilian Amazon.

Regarding conservation units, we obtained a much lower percentage than Indigenous Lands (ITs), with Sustainable Use Units (SUA) having the second-highest percentage, accounting for 17% of chelonians occurrences in these areas. Protected Areas of Full Protection (SPAs) showed a lower occurrence of chelonians at 9%. We obtained few occurrence points with geographic coordinates for SPAs, and it is unclear whether this is due to these areas being difficult to access or having fewer research efforts.

Preserving key reproduction sites, significantly impacted by hydroelectric power plants, is more of a management issue than a sanctuary issue due to the cultural significance of consuming these species. Their distribution within Protected Areas (PAs) and Indigenous Lands (ITs) suggests that the area being protected or not may not be as crucial. There could be external impacts on these areas (such as dam construction) but managing these species in Indigenous Lands is essential for their conservation.

These results highlight areas with higher occurrences that are suitable for establishing PAs, considering the conservation of all animal species, both aquatic and terrestrial. In the case of Indigenous Lands, management efforts can be implemented to improve the survival of these chelonians and raise awareness among traditional communities about the importance of these species for nature and their livelihoods. However, regulatory bodies need to establish appropriate measures to prevent illegal sales and unregulated consumption of these chelonians, particularly during the nesting season.

## Materials and methods

### Study area

**Figure 4:**
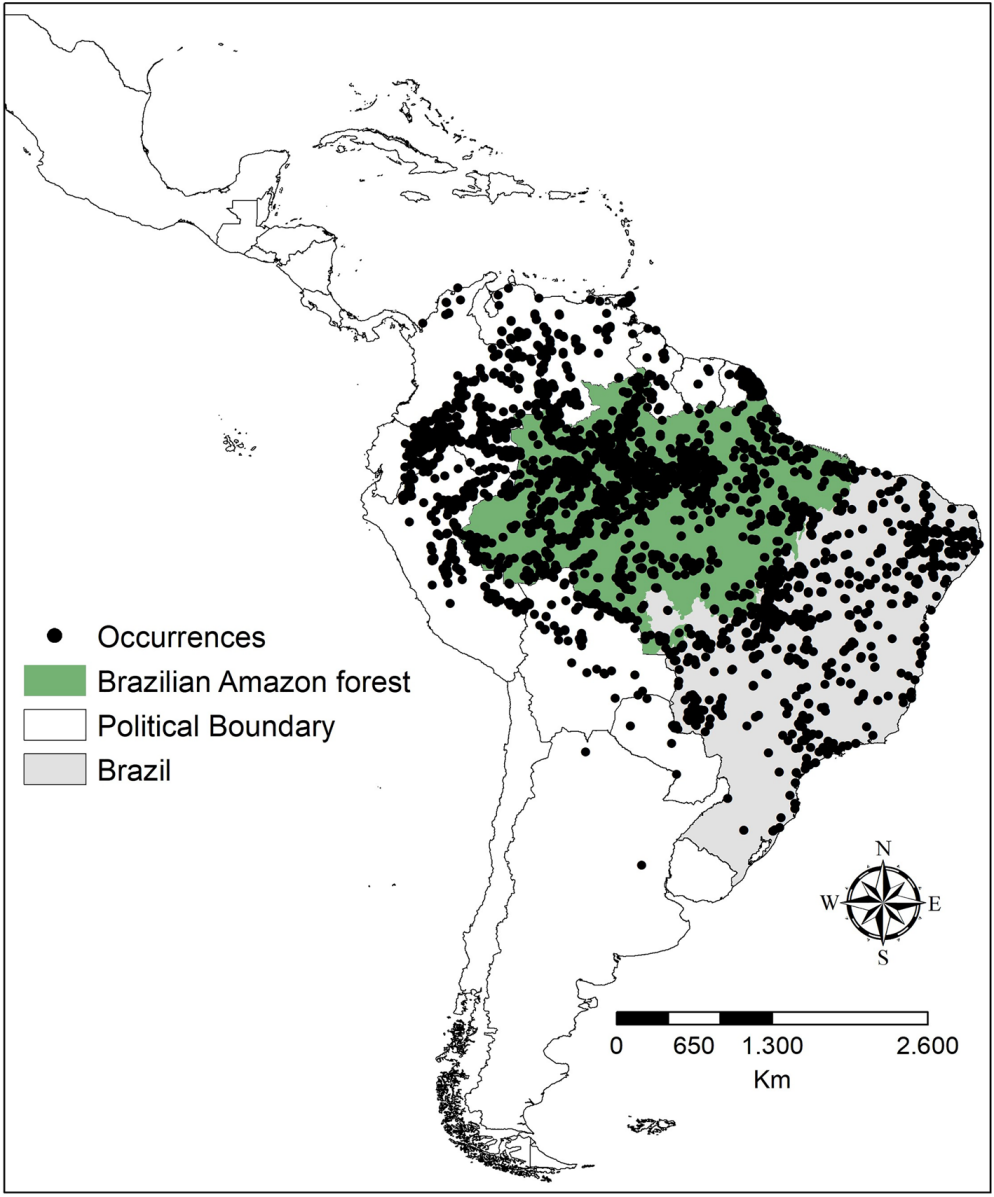
Geographic location of the Brazilian Amazon Basin, indicating the occurrence points of the found chelonians. The blue lines represent the drainage, and the black lines represent the political divisions of South America. To create the figures, the Qgis software version 3.34.3 (https://qgis.org/pt_BR/site/) was used.

The Brazilian Amazon basin covers approximately 3.8 million square kilometers, spanning seven states: Acre, Amazonas, Roraima, Rondônia, Mato Grosso, Pará, and Amapá (Fig. 4). The region has a predominantly tropical rainy climate, characterized by hot and humid conditions, with rainfall concentrated from November to March and a dry period between May and September^62^. The average annual temperature is 27.9 °C during the dry season and 25.8 °C in the rainy season^62^. The soil in the region is a combination of various geological and geomorphological factors with a high sodium concentration, resulting in nutrient-poor soil with a thin layer of nutrients formed by the decomposition of organic matter, leaves, flowers, animals, and fruits^62^.

### Identification of the contribution of each type of unit

To identify the contribution of PAs and ITs to the conservation of Amazonian chelonians species, we created a 0.05° grid considering the extent of the Brazilian Amazon basin as the limit. This grid was superimposed on the species distribution maps and the species presence and absence values were extracted for this grid. Subsequently, the grid cells were superimposed on the map of PAs and ITs, and each cell was classified as SPA and SUA and ITs. To be considered in some of these categories, the cell must have at least 75% of its area within PAs or ITs. Cells that did not meet this criterion or were completely outside PAs or ITs were classified as “unprotected”. After these procedures, we calculated the percentage of the distribution area that was in SPA, SUA, ITs and “unprotected” (outside these protection areas). With these data, we performed a one-way Analysis of Variance (ANOVA) to identify whether some of the categories (SPA, SUA, IT and “unprotected”) have a higher percentage of the species' area of occurrence. Additionally, we evaluated the hierarchical analysis of the contribution, starting with the evaluation of the most restrictive PAs, SPA, and adding the SUA and finally the IT. In this sense, we use a category with the percentage of SPA area, a second category with the percentage of SPA area added to SUA and a third category with the sum of the percentages of distribution in SPA, SUA and IT. In this regard, we used a category with the percentage of SPA area, a second category with the percentage of SPA plus SUA area, and a third category with the sum of the percentages of distribution in SPA, SUA, and IT. This procedure was performed for all chelonians species analyzed and subsequently for species classified by habitat (semi-aquatic, and terrestrial), omnivorous, carnivorous, herbivorous dietary species, and type of environment (generalist, lentic, lotic, and terrestrial), and for each threat level, considering both the IUCN lists.

To assess the difference in importance between protected and unprotected cells, we utilized the Monte Carlo randomization test with 10,000 randomizations. For this, the priority area maps were overlaid onto those of Protected Areas (PAs) and Indigenous Lands (ITs), and each cell was classified as Full Protection (SPA and SUA) and Indigenous Lands. To be considered in one of these categories, a cell needed to have at least 75% of its area within a PA or IT. Cells that did not meet this criterion or were entirely outside PAs or ITs were classified as "unprotected." The average conservation importance (calculated by the Zonation algorithm) was then computed for each class of protected areas (SPA, SUA, and IT), and this value was considered as the observed average importance value. Subsequently, the same number of cells present in each class was randomly selected, and the importance value was calculated, generating the random average importance. This random selection procedure was repeated 10,000 times, and the mean of these values was considered as the randomized importance value. The calculation of the significance value was determined by the number of random values greater than or equal to the observed value divided by the total number of randomizations (10,000). This procedure was repeated for all conservation prioritization maps, considering all classes as "protected," and for each type of PA and IT separately. The PA data were obtained from the Ministry of the Environment (MMA), including municipal, state, and federal levels (http://mapas.mma.gov.br/i3geo/datadownload.htm), and the IT data from the National Indian Foundation (FUNAI, http://www.funai.gov.br/index.php/shape). All procedures were conducted using Species Distribution Models (SDMs), methodologies that cover all biological taxa are not always viable for conservation data. Therefore, we chose to use the algorithms proposed by Pimenta (2022). Currently, these methods are widely accepted for analyzing conservation data, reducing the chances of errors and providing more accurate results^63^. To create the figures, the Qgis software version 3.34.3 (https://qgis.org/pt_BR/site/) was used.

### Species used

We found 8989 points for the 21 turtle species included in the study (Table S01) and (Fig. 4). After data cleaning and removal of points without reference and duplicate points, 3,796 points remained (Table S02, Fig. 4). All models presented AUC and TSS values exceeding 0.9 (Table S01). Brazil is one of the richest countries in turtle species, with 40 species throughout the country. This quantity includes species considered semi-aquatic and terrestrial. For this research, we focused on the Amazon biome, which hosts 24 species, freshwater, for modeling there were 21 valid species and with at least five points of occurrence—19 semi-aquatic, and two terrestrial ^37,39^, these chelonians are classified into habits: semiaquatic, and terrestrial, which are as follows: *Rhinoclemmys punctularia* (perema), *Kinosternon scorpioides* (muçuã), *Acanthochelys macrocephala* (tartaruga-do-pontal), *Chelus fimbriata* (mata-mata)*, Chelus orinocensis* (mata-mata)*, Mesoclemmys gibba* (cágado de poças da floresta), *Mesoclemmys nasuta* (cágado de cabeça de sapo), *Mesoclemmys raniceps* (lalá), *Mesoclemmys vanderhaegei* (cágado-cabeçudo), *Phrynops geoffroanus* (cágado de barbicha), *Phrynops tuberosus* (cágado rajado), *Platemys platycephala* (jabuti macho), *Platemys platycephala melanonota* (charapa), *Rhinemys rufipes* (cágado vermelho), *Peltocephalus dumerilianus* (cabeçudo), *Podocnemis erythrocephala* (irapuca), *Podocnemis expansa* (tartaruga da amazônia), *Podocnemis sextuberculata* (pitiú), *Podocnemis unifilis* (tracajá) two terrestrial, *Chelonoidis carbonarius* (jabuti vermelho) e *Chelonoidis denticulatus* (jabuti amarelo). As for their habitat, they are classified as generalists, lentic, lotic, and terrestrial. All the procedures described were carried out for all species present and considering only endangered species (IUCN). They were divided by habitat (aquatic, semi-aquatic, and terrestrial), environment (generalists, lentic, lotic, and terrestrial), and IUCN classification (Table S02).

### Occurrence data and species modeling

We utilized occurrence points for species found in the Brazilian Amazon basin. The search for occurrence points was conducted from 1900 to September 2022, including all occurrence points located within and outside Brazilian territory. We considered data published in both indexed and non-indexed articles, searched digital databases, and consulted museums and curators. Initially, we searched for occurrence points in digital collections such as SpeciesLink (https://specieslink.net/) and the Global Biodiversity Information Facility (GBIF; www.gbif.org), using species as keywords. For indexed literature, we used the ISI Web of Knowledge and Google Scholar databases with keywords such as "Podocnemididae," "Turtles," and "Testudine." Data with (1) occurrence records lacking dates and (2) records without coordinates were excluded. To minimize overfitting in the models, we filtered the occurrences for each species, avoiding duplicated occurrences and spatial autocorrelation. This procedure performing a Moran’s correlogram (based on the linear distance between points) and identify and remove occurrences with significant autocorrelation, including the duplicated points. The number of unique occurrences of each species is presented in Table S02.

### Environmental variables

In this study, we used hydrological variables, which were combined with climatic, physiographic, soil, and geological variables for modeling aquatic species^64,65^. For freshwater species, we used the Hydroatlas 4 database containing 31 bioclimatic variables related to hydrology, hydrography, climate, soil types, and geology, with high spatial resolution of 1 km^65^. For terrestrial species, we employed the 19 bioclimatic variables available on WorldClim (http://www.worldclim.org)^65^. These variables belong to a group of climatic variables derived from monthly temperature and precipitation values sampled throughout 1960–1990. To reduce multicollinearity in our dataset, a Principal Component Analysis (PCA) was performed^66^ and used the eigenvalues as environmental variables. Next, we selected only the axes that represent an explanation equal to or greater than 95%^67^, using these axes as model variables.

## Algorithms

Models were created using four algorithms: Maximum Entropy (MXD)^68,69^, Random Forest (RDF)^70^, Support Vector Machine (SVM)^71^ and Gausian-Bayesian (GAU)^72^. To reduce uncertainty caused by different algorithms, an ensemble combining the final suitability maps generated by the algorithms was created^73–75^. The RDF and SVM algorithms require species absence data. Therefore, we will create 50 pseudo-absences based on an environmental envelope to allocate pseudo-absences only in locations considered unsuitable for the occurrence of species^76^. In the case of MXT, the models will be adjusted by the differentiation between occurrence records and 10,000 background points randomly sampled throughout the study area, while GAU is for the analysis of static observational time series data, static interventional data, and dynamic data.

To minimize model uncertainties, we considered an ensemble model as the final model^63,77^. This ensemble model involves averaging the suitability values from models where the Jaccard threshold values^63^ were greater than the average thresholds for each species^77^. The Jaccard threshold was selected to minimize omission and commission errors in the models^63^.

Additionally, we imposed spatial constraints on the models to minimize overprediction in distribution models^63,78^. To achieve this, we created a binary occurrence map, where suitability values greater than the Jaccard threshold indicated species presence. We then divided this map into patches (basins) with and without species occurrence. Subsequently, we retained only the basins where the species was predicted and had occurrence records, or basins where the species was predicted and connected to basins with predictions and occurrence points, in the potential distribution map for the species^63^. For partitioning the binary map, we considered two methods: (1) Species with more than 30 occurrence points—map partition using the chessboard method^79^; (2) Species with fewer than 30 points—Random selection of a percentage of points for modeling and another for evaluation, with 70% of points selected for the model and 30% for evaluation^63^. All procedures were carried out using the ENMTML function implemented in the ENMTL package ENMTL^79^ in R environment^80^.

## Model evaluation

The evaluation was performed using Receiver Operating Characteristic (ROC) curves, and the efficiency of each model was assessed through the True Skill Statistic (TSS) analysis, widely advocated as an appropriate discrimination metric that is independent of prevalence^81,82^. TSS is an intuitive method for measuring SDM performance, calculating sensitivity (true positive rate (TPR)) and specificity (true negative rate (TNR)) values, where predictions are expressed as presence-absence maps. This test slightly narrows down the occurrence area, leading to a less inclusive map, considering errors of omission in species distribution (false negative) and commission (false positive), with variation between − 1 and + 1 (Sensitivity + Specificity) to indicate the predictive ability of the models. Models with TSS values close to + 1 reflect good predictive capacity, models with TSS between 0.2 and 0.6 are considered fair and/or moderate, and models with TSS close to 0 and negative values indicate low capacity.

However, TSS values can be misleading when the number of true negatives assigns higher values to species with lower prevalence^83^. To avoid these deficiencies, we propose to focus the evaluation metrics on three components of the confusion matrix: true positives, false positives, and false negatives, neglecting true negatives that could inflate the data. In other words, we seek to maximize true positives and minimize false positives and false negatives in relation to true positives^84^.

### Supplementary Information

Supplementary Figure S1.

Supplementary Figure S2.

Supplementary Figure S3.

Supplementary Table S1.

Supplementary Table S2.

## Data Availability

All data files are in scope of the text or supplementary material.
