# Supplementary figures and images for: Conservation units alone are insufficient to protect Brazilian Amazonian chelonians
Source: Sci Rep. 2024 May 11;14:10827. doi: 10.1038/s41598-024-61722-y (PMC11088686; doi:10.1038/s41598-024-61722-y)

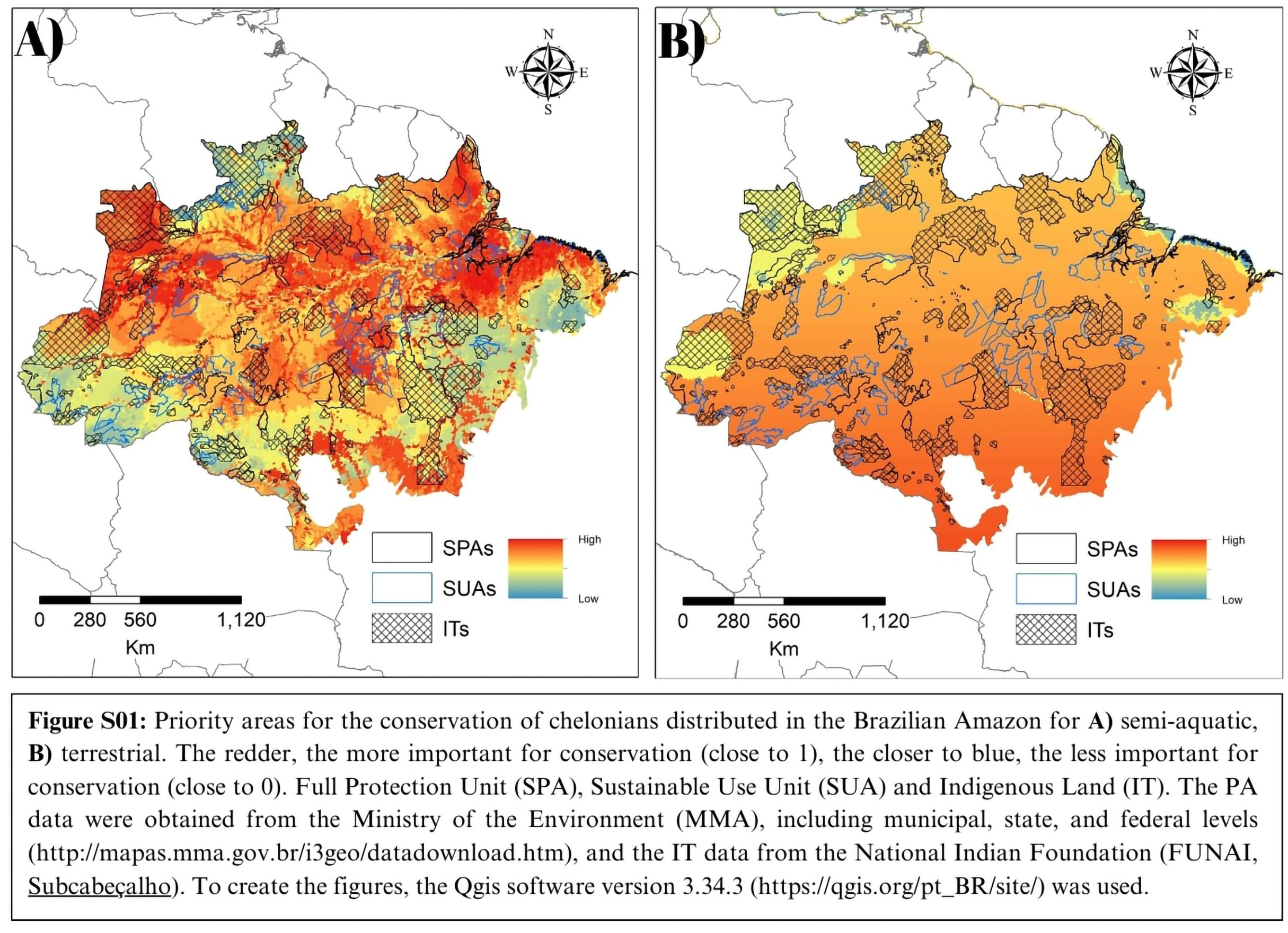

Supplement: Supplementary file 1 — Supplementary Figure S1. [file 41598_2024_61722_MOESM1_ESM.jpg]

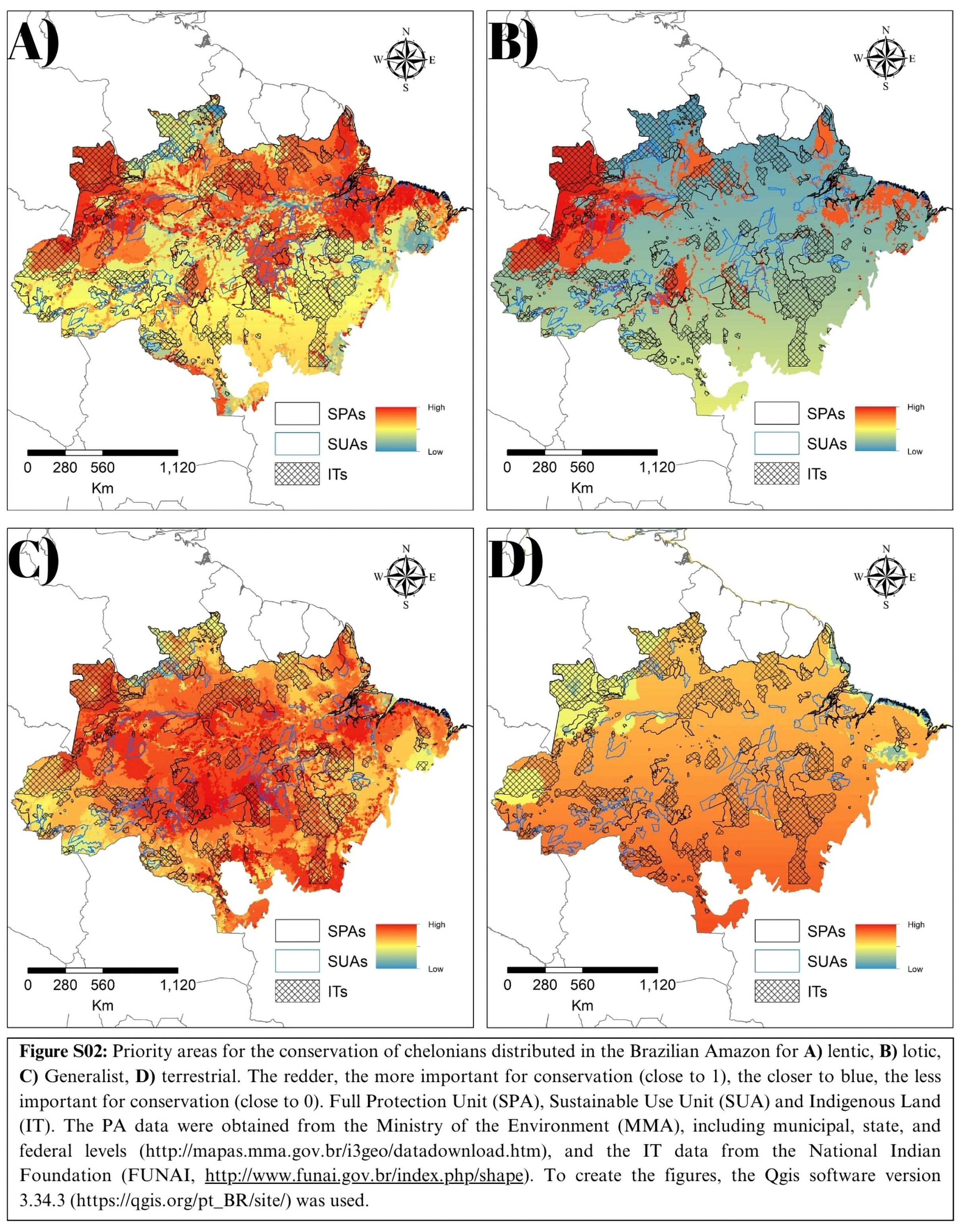

Supplement: Supplementary file 2 — Supplementary Figure S2. [file 41598_2024_61722_MOESM2_ESM.jpg]

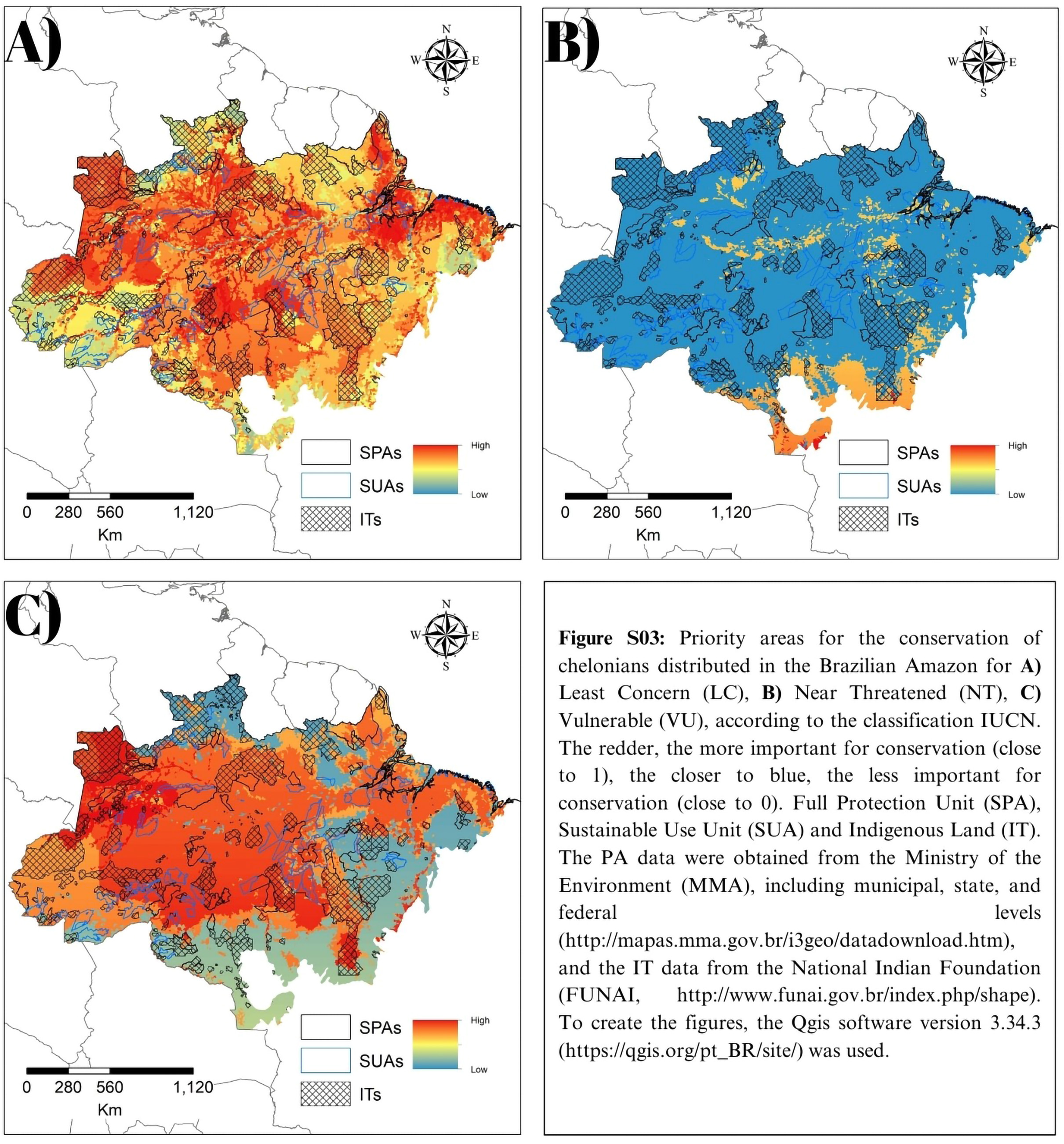

Supplement: Supplementary file 3 — Supplementary Figure S3. [file 41598_2024_61722_MOESM3_ESM.jpg]
